# From genetics to systems biology of stress-related mental disorders

**DOI:** 10.1016/j.ynstr.2021.100393

**Published:** 2021-09-12

**Authors:** Shareefa Dalvie, Chris Chatzinakos, Obada Al Zoubi, Foivos Georgiadis, PGC-PTSD Systems Biology workgroup, Lee Lancashire, Nikolaos P. Daskalakis

**Affiliations:** a South African Medical Research Council (SAMRC), Unit on Risk & Resilience in Mental Disorders, Department of Psychiatry and Neuroscience Institute, University of Cape Town, Cape Town, South Africa; b South African Medical Research Council (SAMRC), Unit on Child & Adolescent Health, Department of Paediatrics and Child Health, University of Cape Town, Cape Town, South Africa; c Department of Psychiatry, McLean Hospital, Harvard Medical School, Belmont, USA; d Stanley Center for Psychiatric Research, Broad Institute of MIT and Harvard, Cambridge, USA; e Department of Data Science, Cohen Veterans Bioscience, New York, USA

**Keywords:** Stress disorders, Traumatic, Genetics, Systems biology, Transcriptome, Epigenomics

## Abstract

Many individuals will be exposed to some form of traumatic stress in their lifetime which, in turn, increases the likelihood of developing stress-related disorders such as post-traumatic stress disorder (PTSD), major depressive disorder (MDD) and anxiety disorders (ANX). The development of these disorders is also influenced by genetics and have heritability estimates ranging between ∼30 and 70%. In this review, we provide an overview of the findings of genome-wide association studies for PTSD, depression and ANX, and we observe a clear genetic overlap between these three diagnostic categories. We go on to highlight the results from transcriptomic and epigenomic studies, and, given the multifactorial nature of stress-related disorders, we provide an overview of the gene-environment studies that have been conducted to date. Finally, we discuss systems biology approaches that are now seeing wider utility in determining a more holistic view of these complex disorders.

## 1 Introduction

Most individuals will experience some form of traumatic stress in their lifetime. According to a global population survey, over 70% of respondents reported exposure to a traumatic event (Benjet et al., 2016), an alarming statistic given that trauma experienced during both childhood and adulthood increases the risk for the development of psychopathology (Mersky et al., 2018; Myers et al., 2015) and physical illness (Basu et al., 2017; Le Carolyn et al., 2013; Suglia et al., 2018). It remains unclear what the exact biological mechanisms are which mediate the association between trauma and negative health outcomes. However, not all who experience a trauma will develop a negative outcome. For instance, while, by definition, a traumatic event is required for the development of post-traumatic stress disorder (PTSD), not all trauma-exposed individuals will go on to develop the disorder, and many will recover quickly. This indicates that certain individuals are at increased risk of developing PTSD, perhaps in part, a result of genetic liability (Cornelis et al., 2010).

Along with PTSD, Major Depressive Disorder (MDD) and Anxiety Disorders (ANX) are commonly referred to as “stress-related disorders” (Smoller, 2016). These disorders have a relatively high lifetime prevalence, ranging between 4 and 29% (Kessler et al., 2005; Koenen et al., 2017), and result in considerable health and economic burden (Greenberg et al., 2015; Koenen et al., 2017; Lepine and Briley, 2011; Olatunji et al., 2007). In this review, we provide an overview of the genomic mechanisms underlying these three disorders by summarising the findings from recent genome-wide association studies (GWAS), and studies of the transcriptome and the epigenome. Given the complex nature of stress-related disorders, we also provide an overview of systems biology approaches that have been implemented to gain a more holistic insight into the mechanisms underlying PTSD, MDD and ANX.

## 2 Genetics of stress-related disorders

**Fig. 1:**
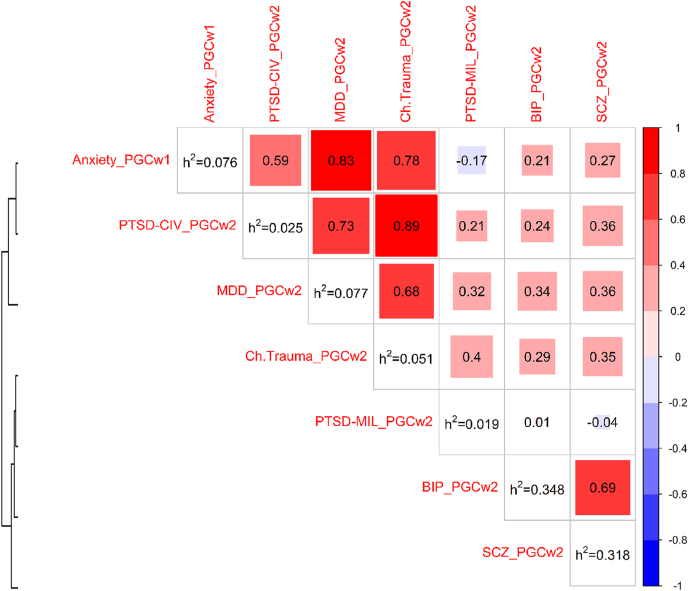
Genetic correlation (*r*_*g*_) using Linkage Disequilibrium Score Regression (LDSC) (Bulik-Sullivan et al., 2015). Heatmap depicting *r*_*g*_ between psychiatric disorders, where red denotes positive and blue denotes negative correlation estimates. The rows and columns of the heatmap are hierarchically clustered based on the correlation coefficients. The heritability (h^2^) estimates based on LDSC are given in the diagonal. Studies: 1) childhood trauma GWAS from Psychiatric Genomics Consortium (PGC) wave 2 (Ch.Trauma_PGCw2) (Dalvie et al., 2020); 2) major depression disorder PGC-GWAS wave 2 (MDD_PGCw2) (Wray et al., 2018), 3) GWAS of posttraumatic stress disorder (PTSD) in 21 European Civilian cohorts part of the PGC-PTSD wave 2 study (PTSD-CIV_PGCw2) (Nievergelt et al., 2019), 4) PTSD-GWAS in 20 European military cohorts part of the PGC-PTSD wave 2 study (PTSD-MIL_PGCw2), and 5) anxiety GWAS from PGC wave 1 (Anxiety_PGCw1) (Otowa et al., 2016a). For comparison, two additional studies are included: schizophrenia (SCZ_PGCw2) (Schizophrenia Working Group of the Psychiatric Genomics et al., 2014) and bipolar disorder (BIP_PGCw2) (Stahl et al., 2019) based on PGC wave 2. Note that h^2^ is not reported on a liability scale. (For interpretation of the references to colour in this figure legend, the reader is referred to the Web version of this article.)

As the name suggests, stress-related disorders typically arise following exposure to an environmental factor such traumatic stress. However, the development of these types of disorders also have genetic contributions. PTSD has a strong genetic component with twin studies reporting heritability estimates from 30% to as high as 72% (Sartor et al., 2012; Stein et al., 2002; True et al., 1993) and SNP-based heritability estimated to be between 5 and 20% (Duncan et al., 2018; Nievergelt et al., 2019). Similar to PTSD, family and twin studies show heritability ranging between 30 and 40% for MDD (Sullivan et al., 2000) and 32–43% for ANX (Hettema et al., 2001) and SNP-based heritability (*h*^*2*^) of ∼9% and ∼7–12%, respectively (Dunn et al., 2017; Howard et al., 2019; Stein et al., 2017). We used LD-score regression score (LDSC) (Bulik-Sullivan et al., 2015) to estimate SNP-based *h*^*2*^ for PTSD (separated by military and civilian cohorts), MDD and ANX (displayed in Fig. 1)**.** MDD and ANX had the highest *h*^*2*^ (i.e., 7.7% and 7.6% respectively), followed by childhood trauma in the Psychiatric Genomics Consortium (PGC)-PTSD cohort (i.e., 5.1%), with PTSD traits having the lowest *h*^*2*^ (i.e., 2.5% for the PGC-PTSD civilian cohorts and 1.9% for the PGC-PTSD military cohorts). LDSC genetic correlation (*r*_*g*_) analyses show that PTSD, MDD and ANX have a considerable genetic overlap (Fig. 1). In the following sections we describe the common and rare variation that have been identified for PTSD, depression/MDD and ANX and also outline the findings from polygenic risk scoring studies for these disorders.

### 2.1 Common variation

Stress-related disorders are thought to have a polygenic architecture with each risk variant having a small effect size (Smoller, 2016). Due to collaborative efforts such as the PGC, well powered GWASs have been instrumental in identifying common variation associated with a range of stress-related disorders (Howard et al., 2019; Meier et al., 2019; Nievergelt et al., 2019). For example, the first study to identify genome-wide significant loci for PTSD was one of the largest multi-cohort GWAS for PTSD (∼30,000 cases and 170,000 controls), comprising military and civilian cohorts [including UK Biobank (UKBB) samples]. Here, six significant hits were identified, four specifically in the European and two in the African-ancestry groups. When functionally annotated, these variants mapped to genes *ZDHHC14*, *PARK2*, *KAZN*, *TMRM51-AS1*, *ZNF813*, LINC02335, *MIR5007*, *TUC338*, LINC02571, and *HLA-B*. Many of these genes had not been implicated in the aetiology of PTSD before, and subsequent gene-set analysis suggested the involvement of pathways of the immune system in PTSD (Nievergelt et al., 2019). Following this, the largest single cohort GWAS for PTSD to date (N > 250,000) was conducted using participants from the US Million Veteran Program (MVP). This study found 3 genome-wide loci (hits were close to the genes *METTL15*, *AUTS2*, *MAD1L1*) for PTSD diagnosis in the European ancestry group. An additional 2 hits were identified in the African ancestry group located in *CNTN6* and near the gene *BMP2*, respectively (Stein et al., 2021). Several more significant hits for quantitative PTSD symptoms such as total PTSD Symptom Checklist (PCL) score, and the symptom clusters hyper-arousal, avoidance, and re-experiencing have also been found (Gelernter et al., 2019; Stein et al., 2021). In a European ancestry group, 3 significant regions were associated with re-experiencing symptoms including *CAMKV*, a region on chromosome 17 in high-LD with *CRHR1*, and *TCF4*; all previously associated with other neuropsychiatric traits and the central stress response (Gelernter et al., 2019). Although there have not been obvious convergent pathways and loci emerging across these large-scale PTSD GWASs, it is worth noting that several of the significant variants from the MVP study were nominally replicated in Nievergelt et al., (2019). In addition, PRS derived from the MVP GWAS summary statistics significantly predicted the variance in the PGC-PTSD study (Stein et al., 2021).

A large GWAS for depression phenotypes (∼250,000 cases and ∼560,000 controls) comprising data from 23andMe Inc., UKBB and the PGC found 102 risk variants for this disorder, 87 of which were replicated in an independent dataset. Pathway and enrichment analyses indicated the importance of synaptic structure, neurotransmission and prefrontal cortex (PFC) regions in the aetiology of depression (Howard et al., 2019). A closer examination of the hits identified from this GWAS showed that 34 of the significant genes have immune-related function (Tubbs et al., 2020). The largest meta-analysis for depression phenotypes, comprising data from the MVP, 23andMe Inc., UKBB, and FinnGen (N = 1,154,267) identified 178 risk loci in Europeans (Levey et al., 2021). A total of 81 of the significant variants were replicated (p < 5 × 10-8) in an independent dataset. Gene ontology analysis revealed significant enrichment of the following biological processes: nervous system development, synapse assembly, and organization. No genome-wide significant hits were identified in the significantly smaller African American sample (n = 59,600) (Levey et al., 2021). Although these two large-scale GWASs were adequately powered to identify replicable loci in European population groups, these hits may not be specific to the core depression phenotype as many of the studies included in the meta-analyses used a broader diagnostic scale. Future studies should assess these risk loci in clinically ascertained MDD cohorts.

For ANX, the largest GWAS to date, consisting of ∼200,000 participants, identified five genome-wide significant loci for Europeans (*SATB1-AS1*, *ESR1*, near LINC01360 and *LRRIQ3*, *MAD1L1*, near *TCEA2*) and one hit for African Americans (near the gene *TRPV*) (Levey et al., 2020). In the UKBB (cases = 25,453, controls = 58,113), five genome-wide hits (located in intergenic regions, *NTRK2*, *TMEM106B* and *MYH15,* respectively) were identified for lifetime ANX disorder. Although none of these significant loci were replicated for anxiety, two of the hits were shown to be associated with neuroticism and major depressive disorder in an independent dataset (Purves et al., 2020). Both of the above-mentioned GWASs found significant LDSC genetic correlation (*r*_*g*_) between ANX and depressive symptoms (*r*_*g*_ = 0.78–0.81). This is unsurprising given the symptom overlap between these two phenotypes and high comorbidity (Lamers et al., 2011; Waszczuk et al., 2014). Another GWAS, comprising ∼12,000 cases with both anxiety and stress-related disorders (including PTSD and depression) and more than 19,000 controls, found the strongest signal for a variant located in *PDE4B* (Meier et al., 2019).

As evidenced by the above-mentioned studies, large sample sizes have led to studies powered enough to detect significant genomic loci of small effect size in stress-related disorders. Moving forward, many of these variants need to be assessed in clinically ascertained cases and be replicated in independent, and ancestrally diverse datasets. In addition, one of the major challenges now is in prioritization of variants in order to elucidate their functional consequences and how these relate to causality. Although the genetic basis for each of these disorders have been researched for many years, the exact causal genes have yet to be identified. We propose that the variants identified from GWAS be functionally characterized by conducting gene expression studies in relevant post-mortem brain tissue and by performing knock-out, knock-in and over-expression studies in relevant cell lines and animal models. Additionally, Mendelian Randomization may be useful in determining statistically whether these variants are causal of stress-related phenotypes.

### 2.2 Rare and structural variation

It is thought that rare variation with large effect sizes are more prevalent in earlier onset, highly heritable disorders such as schizophrenia, and less common in stress-related disorders such as depression, which in turn, has a later onset and is less heritable (Ormel et al., 2019). Nonetheless, there is some evidence to suggest that large copy number variation (CNV) and rare variants play a role in the pathophysiology of stress-related disorders. For example, 53 neurodevelopmental CNVs were found to be associated with self-reported depression in a sample size of more than 500,000 samples (Kendall et al., 2019). Another study examined the contribution of rare variants from exome data in adolescents with PTSD (n = 707). Using a gene-based model (where rare variants for a particular gene were summed), they found four genes associated with lifetime PTSD diagnosis: *MPHOSPH9*, *LGALS13*, *C12orf50* and *SCL2A2* (Sheerin et al., 2019). However, findings have been mixed as another study did not find an association between CNVs and children (n > 12,000) with anxiety or depression (Martin et al., 2019b). Similarly, a study conducted in children (N > 6000) did not find a relationship between burden of CNVs and anxiety (Guyatt et al., 2018). This difference in findings between children and adults may suggest that the effects of CNVs for stress-related disorders are more pronounced from adolescence onwards. Finally, applying re-imputation of rare variants to MDD and PTSD GWAS, very strong association signals were found for rare variants in the genes *H3F3C and PKN2*, respectively (Chatzinakos et al., 2021b). Though the role of rare and copy number variation in stress-related disorders is still not clear, this area is worth investigating further – particularly considering the small number of published studies in this area and the proposed polygenic architecture of these types of disorders.

### 2.3 Polygenic risk scores (PRS)

As mentioned above, stress-related disorders are likely to have contributions from multiple genetic variants, spread across the genome. PRS analysis involves the calculation of a cumulative risk score in a “target” dataset based on the results from a GWAS in a “discovery” dataset. Using PRS in adequately powered samples, it is possible to determine whether variants not achieving genome-wide significance contribute to particular phenotypes of interest, such as schizophrenia (Consortium, 2009). For example, PRS derived from a PTSD GWAS predicted 4.7% of the variance in PTSD onset and 4.4% of the variance in PTSD severity in an independent veteran cohort (Misganaw et al., 2019). Another study found that PRS derived from a MDD GWAS for adults significantly predicted phenotypic variation (diagnosis and severity) in children and adolescents with depression (Halldorsdottir et al., 2019). These findings show that PRS derived from well-powered GWASs are useful in predicting diagnosis and severity of stress-related disorders in independent datasets.

Stress-related disorders show considerable genetic overlap with each other (see above) and this can also be seen with PRS-based analyses. For example, PRS for depression predicted approximately 2.1% of the variance for anxiety in an elderly population group (Demirkan et al., 2011). PRS derived from a GWAS for depression significantly predicted the variance in depression and PTSD in a target dataset (Shen et al., 2020; Waszczuk et al., 2020). Additionally, PRS for anxiety predicted PTSD with a high severity trajectory class, estimated from growth mixture model analysis (Waszczuk et al., 2020). These PRS findings provided further support of shared genetic contributions across stress-related disorders.

PRS may eventually be used as a biomarker enabling *a priori* prediction of a diagnosis or severity of a stress-related disorder. However, several challenges will need to be overcome before the clinical utility of PRS can be fully realised. For example, discovery GWASs need to be well-powered to improve accuracy of prediction and therefore very large sample sizes are required. Current PRS methods make use of GWAS data which only captures the effects of common variation. Newer methods are needed which include the influence of rare variants. Most published GWASs have been conducted in European populations which are not predictive in more diverse ancestry datasets. This threatens to widen the already existent clinical and research disparities between European and non-European population groups. Efforts should be made to have large-scale GWASs for stress-related disorders in more diverse population groups (Ikeda et al., 2021; Martin et al., 2019a).

## 3 Genetic and environmental influences in stress-related disorders

Although PTSD, MDD and ANX have considerable heritability, it is evident that these complex disorders also have environmental influences. In the next sections we provide an overview of the findings from gene-environment interaction, gene-environment correlation, transcriptomic and epigenetic studies. We also highlight some of the common pathways that have emerged from these different approaches.

### 3.1 Gene-environment interactions

Once we have identified genetic risk variants, for example from GWASs, a next step is to investigate the relationship between these markers and the environment. Such types of studies are referred to as gene x environment (G x E) interactions and most studies of this type, particularly in the psychiatric field, consider traumatic life events as the environmental exposure (Musci et al., 2019). Many G X E studies have been conducted for stress-related disorders, mainly using hypothesis-driven approaches, however few of these results have been replicated (Sharma et al., 2016).

PTSD is an ideal example of a G x E interaction, given the ubiquity of an environmental exposure needed for the diagnosis. Based on their biological roles, genes that have commonly been investigated in terms of G x E for PTSD include *FKBP5*, *5-HTTLPR*, and *BDNF* but results have often been somewhat contradictory (reviewed by Sharma et al., 2016). Similarly, for MDD, many candidate-gene based G x E studies have been conducted with variable findings across studies, and a systematic evaluation of previously investigated variants and genes did not find any significant interactions with depression (Van der Auwera et al., 2018). Considering the limited success candidate-gene based studies have had, recent approaches have instead looked to interrogate the entire genome, known as a genome-wide by environment interaction study (GWEIS). A GWEIS of depressive symptoms and traumatic life events (n > 100,000) identified significant interactions for the SNPs rs12789145 (downstream of *PIWIL4*), rs17070072 (located in *ZCCHC2*), rs12000047 and rs12005200 (upstream of *CYLC2*) in two UK-based population cohorts. In addition, the genes *MTNR1B* and *PHF2* were found to be significant in the GWEIS gene-based analysis (Arnau-Soler et al., 2019). A study conducted in an East Asian sample found a significant interaction between rs10485715 (downstream of *BMP2*) and traumatic life events for depression (n = 1112). Another study did not find genome-wide significant interactions between traumatic life events and depression in a Japanese cohort but did find suggestive evidence (p < 5.0 × 10^−6^) for the SNP rs10510057 (located close to *RGS10*) (Otowa et al., 2016b). Although some promising results have emerged from GWEIS, indicating significant interactions between the environment and genetic liability, many of these findings still need to be replicated.

### 3.2 Gene-environment correlation

Even though environmental exposures are not typically thought of as heritable traits, behavioural genetics studies have revealed heritability of many exposures perceived as environmental, such as traumatic life events and peer interactions (Kendler and Baker, 2007). Such heritability is referred to as gene–environment correlation (rGE), defined as the genetic differences in exposure to certain environments (Jaffee and Price, 2008). For example, self-reported childhood trauma has a twin- and SNP-based heritability estimate of ∼6% (Dalvie et al., 2020; Jay Schulz-Heik et al., 2009) (See Fig. 1). Interestingly, childhood trauma and traumatic life events have a significantly high *r*_*g*_ with depressive symptoms (70% and 72%, respectively) (Arnau-Soler et al., 2019; Dalvie et al., 2020) (see Fig. 1). These findings suggest common genetic influences on depression and the report of adverse events experienced throughout the lifetime.

### 3.3 Transcriptomic studies of stress-related disorders

Significant differences in gene expression levels have been observed in individuals who have experienced trauma, compared to the unexposed (Minelli et al., 2018). Thus, large scale expression studies may provide important clues to the biological mechanisms underlying stress-related disorders. The largest blood-based transcriptome-wide mega-analysis of PTSD, comprising 229 cases and 311 controls, found significant differential gene expression changes amongst trauma specific case-control groups. Although relatively few genes were consistently differentially expressed across all comparisons, a higher degree of overlap was seen at the level of perturbed biological pathways. For example, changes were enriched for genes involved in immune pathways, such as cytokine, innate immune and type I interferon, consistent with previous research implicating this system in PTSD (Breen et al., 2018). A twin study (n = 79) found 30 differentially expressed genes associated with a lifetime history of MDD in blood tissue. Functional enrichment analysis revealed that these genes are involved in biological processes such as “positive regulation of cytokine secretion” and “regulation of response to stress” (Zhu et al., 2019). Similarly, 631 genes were differentially expressed amongst male cases (n = 43) with anxiety symptoms compared to controls (n = 69) and these genes were enriched for immune-related functions (Wingo and Gibson, 2015).

All of the above transcriptomic studies were conducted using peripheral tissues. Due to the nature of psychiatric disorders, brain tissue is the most relevant tissue in terms of aetiology (Vornholt et al., 2019) . Thus, studies conducted in post-mortem tissue may provide a more accurate description of gene expression changes that occur in stress-related disorders. However, working with post-mortem tissue has several challenges including the logistic requirements, ethical and cultural barriers (Vornholt et al., 2019). Below we highlight some of the post-mortem expression studies that have been conducted for stress-related disorders.

In post-mortem PFC tissue for PTSD, a significant down-regulation of interneuron transcripts and immune related genes were observed in two transcriptomic studies (52 cases and 46 controls; 107 cases and 109 controls, respectively) (Girgenti et al., 2021; Jaffe et al., 2021). By integrating expression with GWAS data, one study also found the interneuron synaptic gene *ELFN1* to be risk gene for PTSD (Girgenti et al., 2021). In the larger post-mortem study, that included tissue from both PFC and amygdala, distinct genes were differentially expressed across PTSD and MDD (n_PTSD_ = 107, n_MDD_ = 109, n_controls_ = 109) but a high number of overlapping genes between the two disorders were also found. Network analysis of these differentially expressed genes showed that dysregulated neuroinflammation and immune signalling are involved in both PTSD and MDD (Jaffe et al., 2021). Interestingly, gene expression signatures in mouse stress models had significant overlap with gene expression signatures in post-mortem brain tissue in humans with MDD (26 cases and 22 controls) (Labonte et al., 2017; Scarpa et al., 2020). It is worth noting that most of the transcriptomic studies described above had quite small sample sizes and have not been replicated. Therefore, these results should be treated with caution as these may be false positives.

### 3.4 Epigenome-wide association studies (EWAS)

Epigenetics is the study of heritable changes in gene function that are not due to changes in the DNA sequence (Dupont et al., 2009). The interrogation of epigenetic modifications, such as DNA methylation, provides the opportunity to gain some insight into the effects of environmental influences on the genome (Martin and Fry, 2018). As with gene expression studies, individuals exposed to trauma show changes in their methylation levels compared to the unexposed (Dalvie and Daskalakis, 2021; Houtepen et al., 2016; Neves et al., 2019). The largest EWAS for PTSD (1896 cases and trauma-exposed controls), comprising both military and civilian cohorts, found 4 significant CpG sites located within the gene *AHRR,* a region commonly associated with smoking (Smith et al., 2020). Another PTSD EWAS (378 cases and 135 controls) found a CpG site located in the gene *G0S2* associated with this disorder. This finding was replicated in an independent cohort (Logue et al., 2020) and was supported by blood and brain expression patterns described in an animal model of PTSD based on predator stress (Daskalakis et al., 2014). The authors also tested the effects of their top ten methylation findings in PFC tissue and found that a CpG site in the gene *CHST11* was associated with PTSD (Logue et al., 2020). Although a slightly smaller EWAS with 473 study participants was underpowered to detect genome-wide significant hits, it found that PTSD was associated with biological pathways regulating neuronal signalling, inflammation and aspects of physical health (Kuan et al., 2017). A longitudinal EWAS for PTSD which meta-analyzed 3 male military cohorts (123 cases and 143 controls) found 3 epigenome-wide significant CpGs-one of which was intergenic while the other 2 were located in genes *MAD1L1* and *HEXDC*. In addition, 12 significant differential methylated regions (DMRs) were found; 4 of these regions were located in the human leukocyte antigen (HLA) and 1 was situated in *MAD1L1* (Snijders et al., 2020). Interestingly, as mentioned above in the “common variation” section, a SNP in *MAD1LI* was associated with PTSD in the largest PTSD GWAS (Stein et al., 2021).

One of the largest EWAS studies for MDD (n = 1132) found suggestive evidence (p < 1 × 10^−5^) of significance at several loci spread across the genome in blood, with overlapping findings observed in brain tissue (Aberg et al., 2020). A study of 79 monozygotic twin pairs found an additional 39 differentially methylated regions (DMRs) associated with a lifetime history of MDD in blood tissue. These regions were enriched for pathways involved in stress-activated protein kinase signalling, neuronal apoptotic processes, insulin receptor signalling, mTOR signalling, and nerve growth factor receptor signalling. Encouragingly, 10 of the identified DMRs in blood were significantly associated with MDD in brain tissue (Zhu et al., 2019).

Several EWASs have been conducted for ANX. A total of 230 differentially methylated regions were found in 299 monozygotic twin pairs discordant for anxiety. These regions mapped to genes previously associated with stress-related phenotypes (Alisch et al., 2017). Another study for ANX identified 40 CpG sites significantly differentially methylated between cases (n = 48) and controls (n = 48). Pathway analysis of these sites found an enrichment of genes involved in “positive regulation of lymphocyte activation” (Shimada-Sugimoto et al., 2017). In a European sample, one genome-wide significant signal in the promoter region of the *HECA* gene was associated with ANX, specifically in females (49 cases and 48 controls). This locus was replicated in an independent sample (Iurato et al., 2017). In a population-based cohort (n = 1522), severe anxiety was associated with increased methylation at a CpG site located in the gene *ASB1*, a gene involved in cytokine regulation (Emeny et al., 2018). As with the transcriptomic studies, the findings from these EWASs for stress-related disorders should be treated with caution given the generally small sample sizes and lack of replication in most instances.

### 3.5 Common pathways

When considering the findings from GWAS, transcriptomic and EWAS studies, there appears to be overlapping loci and pathways across all the genomic “layers” for each stress disorder. For example, the gene *MAD1L1* (a component of the mitotic spindle-assembly checkpoint) was shown to be a risk factor for PTSD in GWAS and EWAS studies (Snijders et al., 2020; Stein et al., 2021), and has also been shown to be associated with ANX (Levey et al., 2020), revealing shared pathways across the different stress-related disorders, in line with their genetic overlap.

Immune- and/or glucocorticoid related pathways have been consistently implicated in all three stress-related disorders (Breen et al., 2018; Daskalakis et al., 2016; Jaffe et al., 2021; Sha and Banihashemi, 2020; Wingo and Gibson, 2015). Pathway and gene-set analysis of GWAS data has also implicated the immune system in the aetiology of PTSD (Nievergelt et al., 2019). This is in agreement with previous studies which found a link between stress-related disorders and auto-immune diseases (Song et al., 2018) and elevated immune biomarkers (Yang and Jiang, 2020). Convergence of these pathways between blood and brain has been reported in animal models of individual differences in the behavioural response to traumatic stress (Daskalakis et al., 2014; Lori et al., 2018). Though the immune pathway overlap between stress related disorders has been now described at the blood and brain level, the sample sizes remain relatively small. Additional caution in interpretation is warranted based on gene annotation biases noted in the widely-used databases used for these analyses (Haynes et al., 2018).

## 4 Systems biology approaches

As demonstrated by the studies reviewed in the previous sections, the underlying aetiology of stress-related disorders is likely the result of a complex interplay between various molecular systems and the environment. To delineate this, a more comprehensive approach that integrates different omics layers with environmental exposures may be the next logical step.

As opposed to reductionist approaches, systems biology aims to interrogate pathophysiology in a more holistic manner, utilising genetic data at different levels which incorporates information on environmental influences (via the use of methylation data and its relationship with gene expression). Systems-biology type studies usually involve a large number of data points and therefore need to be adequately powered to avoid the generation of false positive and negative results. Below we describe studies using systems biology-based methods to further understand PTSD and MDD. We have not included details on studies published for ANX as Mufford et al. (2020) provides an extensive review of systems biology approaches specifically for this disorder.

### 4.1 Multi-omic integration

For stress-related disorders, most studies have focused on single omics methods, e.g. EWAS which is used to determine the effects of the environment i.e. traumatic stress exposure, on the genome. However, the underlying aetiology of stress-related disorders is highly likely a result of a complex interplay between common DNA sequence variation, gene expression and epigenetic status. The proposed model for this interaction has been that DNA sequence variation affects methylation, which in turn has an effect on gene expression (Bell et al., 2011; van Eijk et al., 2012). However, it is also known that interactions between the three modalities are complex and may include additional influencing factors such as the environment.

To assess functionality of significant variants identified from GWASs is to conduct expression quantitative trait loci (eQTL) and methylation QTL (meQTL) analyses. An eQTL and meQTL is a variant that predicts variation in gene expression and methylation, respectively (Bell et al., 2011; Nica and Dermitzakis, 2013). For example, an intronic PTSD risk variant, rs363276, located in *SLC18A2*, is an eQTL significantly associated with decreased expression of the genes *SLC18A2* and *PDZD8* in dorsolateral PFC (Bharadwaj et al., 2018). Further, the PTSD risk variant rs717947, was shown to be an meQTL (Almli et al., 2015). In a study investigating depression, 64 significant and replicated meQTLS were identified in blood. They also observed higher methylation at a CpG site located in the *HACE1* promoter and lower *HACE1* expression in post-mortem brain tissue in patients with MDD compared to controls (Ciuculete et al., 2020).

Transcriptomic imputation or genetically regulated-gene expression is an alternate way to assess the relationship between risk variants and gene expression by conducting the so-called Transcriptome-wide association studies (TWAS) (Chatzinakos et al., 2021a). One such study found that expression of two genes were dysregulated in PTSD, upregulation of *ZNF140* in whole blood and downregulation of *SNRNP35* in PFC, which were validated in cell culture, mouse PFC, and blood expression from PTSD cases (Huckins et al., 2020).

**Fig. 2:**
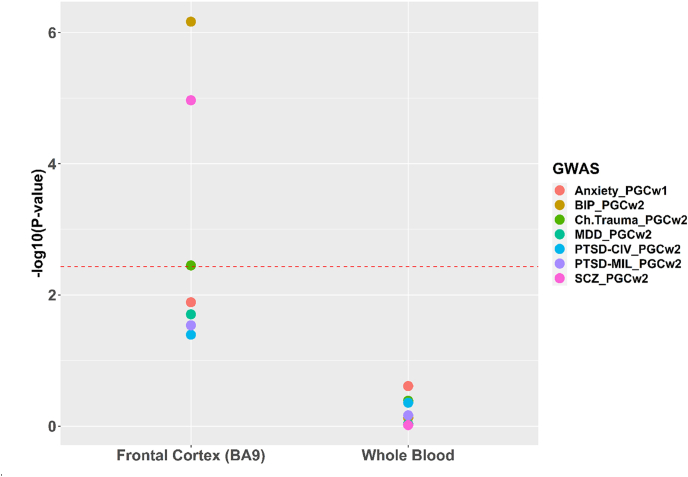
Partitioned heritability analyses of the GWAS included in Fig. 1, using stratified Linkage Disequilibrium Score Regression (Finucane et al., 2015), on the basis of highly expressed genes in Genotype-Tissue Expression (GTEx) dorsolateral prefrontal cortex Brodmann area 9 (BA9) and whole blood tissues. P values are provided on the Y-axis, while the red dashed line denotes a P = 0.003571429 corresponding to a Bonferroni significance (based on 14 tests: 7 traits x 2 tissues). (For interpretation of the references to colour in this figure legend, the reader is referred to the Web version of this article.)

**Fig. 3:**
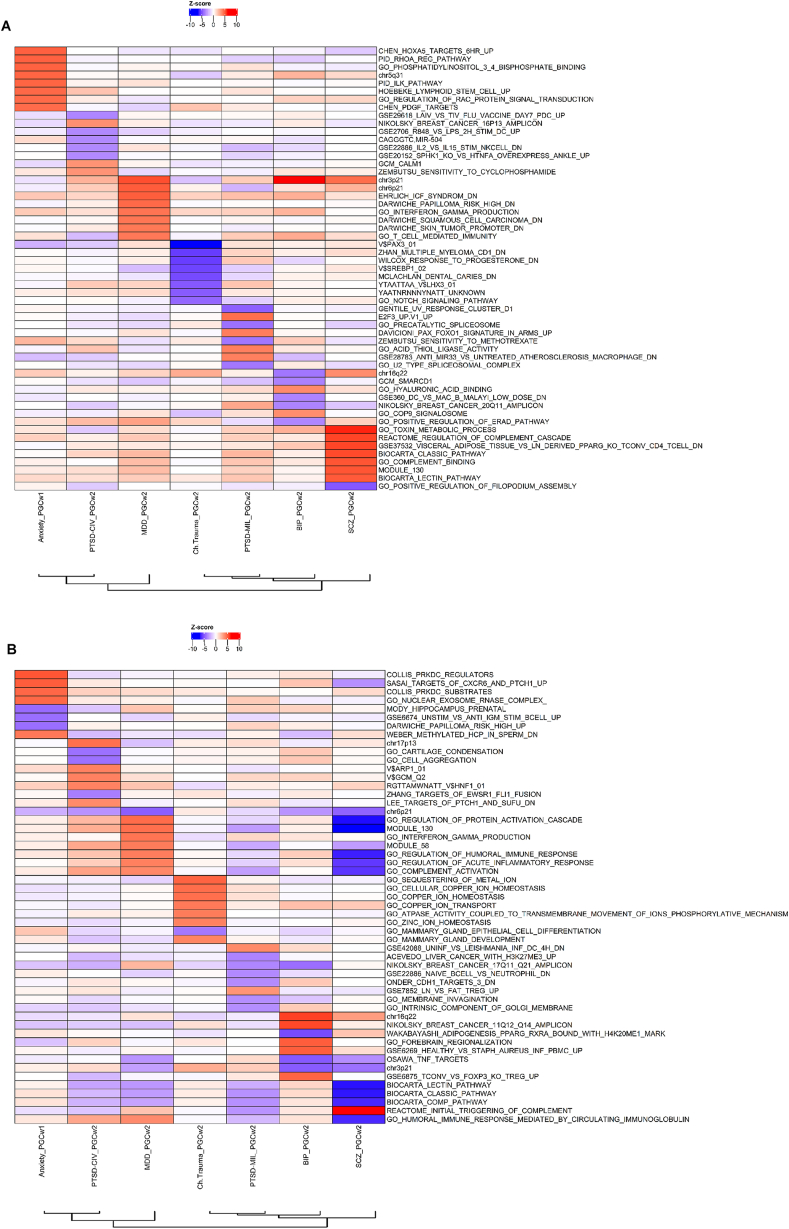
Pathway analyses of GWAS studies included in Fig. 1**(a)** Pathway analysis based on TWAS statistics of Brodmann area 9 (BA9) brain tissue using JEPEGMIX2-P. Heatmap depicting pathway z-scores of the 10 top pathways of each psychiatric disorder. Red denotes up-regulated pathways while blue denotes down-regulated pathways. The order on the x-axis is according to the cluster dendrogram presented in Fig. 1 while the y-axis are ordered based on the absolute pathway z-scores across all the traits. **(b)** Pathway analysis based on TWAS statistics of blood tissue using JEPEGMIX2-P. Heatmap depicting pathway z-scores of the 10 top pathways of each psychiatric disorder. Red denotes up-regulated pathways while blue denotes down-regulated pathways. The order on the x-axis is according to the cluster dendrogram presented in Fig. 1 while the y-axis is ordered based on the absolute pathway z-scores across all the traits. (For interpretation of the references to colour in this figure legend, the reader is referred to the Web version of this article.)

In Fig. 2, using stratified LDSC (Finucane et al., 2015), we show that PFC tissue gene expression explains/partitions heritability of stress-related disorders in the PGC studies. Similar analyses in the MVP study (n = 220K) (Gelernter et al., 2019; Girgenti et al., 2021; Stein et al., 2021) confirmed the partitioning heritability estimates and predicted alterations in 17q21.31 genes (*CRHR1* and *MAPT*). Using the JEPEGMIX2-P, a TWAS pathway analysis tool, the pathways involved are shown in Fig. 3a and b (Chatzinakos et al., 2020) for Brodmann area 9 (BA9-frontal cortex brain tissue) and blood, respectively. One of the most significantly up-regulated pathways in MDD is for “*T-cell mediated immunity*” in brain and *“regulation of acute inflammatory response*” in blood. For PTSD, the *“CD4 Thymocyte vs Naïve CD4 Tcell_ Adult_blood_DN*” was down-regulated in BA9 tissue (Fig. 3a).

In addition to genomic data, other sources of data such as the proteome and neuroimaging have also proved invaluable. A recent study integrating gene expression and proteomic data was able to replicate gene expression in protein levels and prioritized the proteins CCND3, TXND5 and TRI26 as potential biomarkers of cognitive deficits in patients with MDD (Schubert et al., 2018). By the integration of transcriptome signatures and brain volume variation in MDD (n ∼ 6000), Sha and Banihashemi (2020) showed that grey matter volume was associated with transcriptome profiles enriched for synaptic transmission, metabolism, immune processes and transmembrane transport. Further, a study integrating GWAS, dorsolateral PFC eQTL and enhancer-promoter physical link data identified the gene *DCC* as being high risk for the development of depression (Li et al., 2020). A similar study identified *FLOT1* as a novel MDD risk gene by integrating significant associations from an MDD GWAS (N = 480,359) with brain eQTLs (Zhong et al., 2019). A total of 99 potential risk genes for depression were identified by leveraging tissue-specific eQTL information. Of these 99 genes, a novel association was found with *C4A*, a gene previously implicated in schizophrenia (Gerring et al., 2019).

Despite several challenges and limitations (for e.g. variable and noisy omics datasets, non-interoperable software and methods, limited pathway and visualisation tools, and high costs (Pinu et al., 2019), multi-omic integration have provided us with some insight into the biology underlying stress-related disorders. However, the exact causal risk factors are largely unknown. By employing techniques such as Mendelian Randomization, which quantifies causality between a risk factor and a disease outcome by using SNP data as an instrumental variable, we may begin to delineate causal relationships (Pasaniuc and Price, 2017).

One of the most important advances in the analysis of complex systems has been the development of computational and analytical methods to use high-dimensional data to understand biological systems in an unbiased manner. For example, microarray and RNA-seq based methods can assess gene expression for tens of thousands of RNA species simultaneously. Network biology provides a framework for representing pair-wise interactions among proteins, genes, gene products, metabolites and other molecular and higher-order species (Zhang and Horvath, 2005). These gene networks represent interactions among genes, and their analyses can uncover functional organization of genes (Carlson et al., 2006), the association of genes and pathways with disease-related phenotypes (Emilsson et al., 2008), as well as biomarkers, and potential drug targets that play a key role via multiple signalling pathways (Emig et al., 2013). Integration of these causal networks with information on genetic variation such as GWAS signals and environmental factors (epigenetic modifications), which act as systematic perturbants of gene interaction networks, allows for the understanding of causality and construction of causal networks (Zhu et al., 2004), that may in turn lead to the identification of key driver genes, or master regulators that lead to disease (Chen et al., 2008).

The human brain is comprised of diverse cell types with specialized functions. Psychiatric symptoms implicate both neuronal and glial cell types, requiring a systematic view of cell type-specific alterations to recognize the molecular changes (Manji et al., 2001, Yamamuro et al., 2015). Gene regulation biology seeks to identify cell type-specific transcription linked to distinct functions. To measure the transcriptome at the cell type level, methods based on fluorescence activated cell sorting (FACS) require millions of cells (Srinivasan et al., 2016; Zhang et al., 2016). Single-cell transcriptomics enables *de novo* deconvolution and transcriptomic profiling of cell types of complex tissues (Darmanis et al., 2015). For frozen brain samples, in which cells are difficult to isolate, single nucleus RNA sequencing (snRNAseq) has emerged as a robust method of assessing a cell's transcriptome through the use of isolated nuclei (Krishnaswami et al., 2016; Lake et al., 2016), with a high concordance between whole-cell RNA and nuclear RNA transcriptomes (Lake et al., 2018). The first study using this technology has revealed distinct expression profile changes in deep layer cortical neurons and in immature oligodendrocyte precursor cells of the dorsolateral PFC in MDD (Nagy et al., 2020).

### 4.2 Machine learning approaches

Machine learning (ML), employing statistics and computer science, is the process by which computers are able to learn from available data (Deo, 2015). ML techniques have been applied to genomic data in order to leverage the abundance of information captured by these technologies in order to select features that enable prediction of several psychiatric disorders (Bracher-Smith et al., 2021).

ML comes in various forms and can be conceptually divided into supervised learning and unsupervised learning. For supervised learning, the learning goal is to create a mapping function between input and output variables (Hastie et al., 2009). Here, output variables, or labels, are considered the ground truth on which to build a model. Different modelling techniques have various levels of transparency on how different inputs are deployed and weighted. For example, artificial neural networks and deep learning models often contain hidden layers. Therefore these models do not lend themselves to easy interpretation, a common requirement in the biological sciences. However, even with this potential drawback, they have shown promise in analyzing genomic datasets (Arloth et al., 2020; Chen et al., 2019; Min et al., 2017; Tranchevent et al., 2019). Deep learning has enabled efficient multimodal neuroimaging fusion, capitalizing on the strength of each modality (Maglanoc et al., 2020; Plis et al., 2014; Sui et al., 2012; Zhang et al., 2020). A convolutional neural network for co-expression was able to predict transcription factor targets, identify disease-related genes and perform causality inference in one framework (Yuan and Bar-Joseph, 2019). Deep learning was adopted with multivariate GWAS to discover disease/trait-associated SNPs with a shared effect on a certain chromatin features in a relevant tissue (Arloth et al., 2020). Approaches such as Random Forest produce decision trees that allow for a better understanding of the relative importance and relationships among variables. Transfer learning repurposes models trained within other domains by adapting the models on input-output pair. One of the limitations in stress related disorders is the lack of data for modelling and transfer learning from other domains could boost these discoveries. For example, transfer learning was used to diagnose PTSD (Banerjee et al., 2019) and predict depression (Rutowski et al., 2020) using pre-trained speech models. In genomics, convolutional networks were able to extract high-level features from other tumor-type samples that could improve lung cancer progression prediction (López-García et al., 2020). Finally, regression tasks quantitatively estimate continuous variables rather than membership of specific groups. For example, researchers used deep learning to successfully predict clinically relevant depression severity score from audio, video, and text inputs (Yang et al., 2017). In a transcriptomic study, blood miRNAs combined with multimodal imaging fusion has shown its potential to disentangle brain mechanisms in MDD (Qi et al., 2018).

In unsupervised learning, the goal is to identify the latent structure of the dataset, i.e. a self-organization of data based on similarities or signatures within the data in order to identify non-random patterns which could be used to draw inferences (Hastie et al., 2009). Therefore, the task of partitioning a heterogenous population into more homogenous subgroups often involves unsupervised learning techniques, since the ground truth i.e., the subgroups, are not known *a priori* and the aim here is to identify them. Perhaps the most recognized approach to partitioning data in this way is cluster analysis. Here, the optimization function of each clustering algorithm seeks to identify the optimal partitioning which minimizes the within-cluster similarity while maximizing between-cluster dissimilarity. In practice, one common hurdle in applying these techniques is that of choice. Different approaches for cluster analysis exist, each with a plethora of definitions for distance measure (e.g. Euclidean, cosine, Manhattan distances, average, maximum, minimum inter-cluster distance, to name a few) and additional optimization functions give rise to a large number of clustering algorithms (Xu and Tian, 2015), each resulting in a different solution. Hierarchical clustering (Johnson, 1967) is a type of unsupervised learning algorithm that generates nested groupings organized in hierarchy. This type of clustering is useful when the number of groups are not known.

The accumulation of electronic health record data and the generation of large amounts of data from imaging and -omics studies have sparked the use of unsupervised learning for diagnosis, prediction of disease outcomes and to stratify patient subpopulations. Recently, (Drysdale et al., 2017)used resting-state functional magnetic resonance imaging (fMRI) data of MDD-diagnosed individuals to identify subpopulations. The study identified four biotypes which showed differential treatment response to transcranial magnetic stimulation. However, these results did not replicate in an independent study (Dinga et al., 2019). The lack of replication, apart from highlighting the general issue of reproducibility in human neuroscience research (Poldrack, 2019), could be attributed to different factors: 1) the poor reproducibility of resting-state fMRI measures due to sample size, duration of scan and hardware configuration (Noble et al., 2017, 2019); 2) the lack of the genetic component in resting-state fMRI [i.e., low *h*^*2*^ demonstrated in the large UKBB dataset (Elliott et al., 2018; Smith et al., 2021)], indicating sensitivity to environment; and 3) the difficulty of obtaining consistent MDD results in the resting-state fMRI (Xia et al., 2019).

Using a genetic approach, Arnedo et al. (2015) used SNPs to identify schizophrenia subpopulations (Arnedo et al., 2015). After filtering out SNPs with low Pearson's R correlation (p > 0.5) to schizophrenia symptomatology, they implemented Non-Negative Matrix Factorization twice, on patient-SNP and patient-symptom data, generating two sets of clusters, “patient-SNP” clusters and “patient-symptom” clusters. They then mapped SNP-clusters to phenotypic clusters using hypergeometric testing and identified 8 putative schizophrenia subtypes enriched for different, albeit potentially overlapping, sets of SNPs.

Michopoulos et al. (2020) used latent growth mixture modelling to delineate disease outcome trajectories from PTSD symptom severity data, both in the acute setting and at 1,3,6 and 12 months post-trauma, to identify chronic, resilient, and recovering PTSD subgroups. Interestingly, the chronic trajectory was characterized by a hypo-inflammatory (lower TNFα and IL-1β) phenotype in the acute setting, showing the potential of inflammatory profiling as biomarkers for stratification of at-risk individuals (Michopoulos et al., 2020). Finally, canonical correlation analysis has been used to identify epigenetic biotypes of PTSD based on the correlation of DNA methylation and physical, psychological and dissociation characteristics (Yang et al., 2020).

Other efforts to parse the heterogeneity of mental disorders are focused on building models of normal brain from biological, behavioral, and demographic data. Normative modeling can build a normative range of brain responses and assign risk scores at the subject level, based on deviation from the norm (Marquand et al., 2016). The brain age (BrainAge) approach uses ML methods to build models of age prediction from healthy control subjects. Those models are later used to predict age from clinical groups (Al Zoubi et al., 2018; Cole and Franke, 2017). The difference between chronological age and predicted age, also known as BrainAge score, is linked to mental disorders (Han et al., 2020). Normative modeling can be more informative over other ML approaches since it can offer biological interpretation of the results by linking known brain responses to measured cognitive or behavioural variables.

ML can substantially advance the detection, understanding, and prediction of mental disorders. However, there are several considerations in using ML to avoid overfitting, data leakage and enhance the generalizability of ML models (Rosenberg et al., 2018; Scheinost et al., 2019). Additionally, one should consider proper methods for handling the high dimensionality of -omics data (Bermingham et al., 2015). All algorithm and parameters selection, including any dimensionality reduction, should be done within the training set only. Reporting of the performance should mention the source of evaluation (training or testing set), the number of samples, the number of and distribution of classes for the classification, and any data assumptions. ML methods, including deep learning, are end-to-end approaches and appear as "black boxes", where they transform raw input data (e.g., genetics, neuroimaging) into compact output like membership probability to a group or single score outcome. Thus, analysis strategies using ML should consider the interpretation of the adopted ML approaches. Luckily, there are several efforts in introducing interpretable ML. These methods rely on quantifying the feature contribution on the outcomes such as the Shapley Additive Explanation (Lundberg et al., 2020), LIME (Ribeiro et al., 2016), and Deep Learning Important FeaTures (DeepLIFT) (Shrikumar et al., 2016).

Taken together, linear methods offer a transparent and direct interpretation of the results. Deep learning models are particularly useful when the there is a complex data structure with topological and other relationships, multimodal input or outputs, or when knowledge sharing is available (Richards et al., 2019; Vogt, 2018).

## 5 Conclusions

Genomic studies have provided encouraging clues to the biological mechanisms underlying stress-related disorders and have highlighted the importance of investigating diverse ancestry groups. In addition, many of the GWAS, expression and epigenetic findings have not been replicated and, the functional consequences of the identified loci largely remain unknown and need to be determined. The exact mechanism by which environmental exposures interact with the genome is also still unclear. A systems biology approach to modelling these data may begin to disentangle these complex relationships and provide evidence of the causal mechanisms underlying disease pathophysiology by identifying the multi-dimensional structure in the data, and the signalling pathways and molecular networks that are perturbed during disease progression. To date, this multi-omic integration of different "omes" across tissue types have prioritized novel loci for PTSD and MDD that warrant further investigation, and these loci may pave the way in identifying new targets for these complex disorders of the brain.

## Funding sources

This work was supported by 10.13039/100000025NIMH R21MH121909 to NPD and 2019 SPARED Center Seed Grant (through 10.13039/100000025NIMH P50-MH115874) to CC. CC and NPD were supported by R01MH117292, P50MH115874 and R01MH106595. NPD was supported by a 2018 NARSAD Young Investigator grants from 10.13039/100000874BBRF, a Jonathan Edward Brooking mental health research fellowship from 10.13039/100006694McLean Hospital, and an appointed KL2 award from 10.13039/100007299Harvard Catalyst | The 10.13039/100007299Harvard Clinical and Translational Science Center (NCATS KL2TR002542, UL1TR002541).

## CRediT author contribution statement

**Shareefa Dalvie:** Conceptualization, Writing – original draft, revision, review & editing. **Chris Chatzinakos:** Formal analysis, Writing – original draft, revision. **Obada Al Zoubi:** Writing – original draft, revision. **Foivos Georgiadis:** Writing – original draft, PGC-PTSD Systems Biology workgroup: Resources. **Lee Lancashire:** Writing – original draft, **Nikolaos P. Daskalakis:** Conceptualization, Writing – original draft, revision, review & editing.

## Declaration of competing interest

LL is employed by Cohen Veterans Bioscience Inc., a nonprofit public charity research organization. In the past 3 years, NPD has held a part-time paid position at Cohen Veterans Bioscience, has been a consultant for Sunovion Pharmaceuticals and is on the scientific advisory board for Sentio Solutions for unrelated work. The other authors report no biomedical financial interests or potential conflicts of interest.
